# Editorial: Zebrafish as a model for human congenital disorders

**DOI:** 10.3389/fcell.2023.1235580

**Published:** 2023-06-20

**Authors:** Megha Kumar, Cheol-Hee Kim, Neha Singla

**Affiliations:** ^1^ CSIR- Centre for Cellular and Molecular Biology (CCMB), Hyderabad, India; ^2^ Academy of Scientific and Innovative Research (AcSIR), Ghaziabad, India; ^3^ Department of Biology, Chungnam National University, Daejeon, Republic of Korea; ^4^ Department of Biophysics, Panjab University, Chandigarh, India

**Keywords:** developmental biology, zebrafish, zebrafish disease models, clustered regularly interspaced short palindromic repeat (CRISPR)/CRISPR associated protein 9 (Cas9)-mediated genome editing, embryogenesis

Zebrafish (*Danio rerio*) is a widely used model organism to study embryogenesis, complex diseases like cancer, physiological processes of ageing and a spectrum of congenital disorders such as microcephaly, neurological diseases and cardiovascular abnormalities.

The zebrafish genome has been completed and large number of genetic mutants are available. Many knockout mutant and transgenic lines are available to identify novel genes during embryogenesis and used as models to study pathophysiology of various human congenital diseases. Further, the zebrafish is widely used for large, high throughput screening of potential therapeutic compounds. The zebrafish has an extensive genetic toolkit and technological advances like optogenetics, single-cell RNA-seq analysis, CRISPR/Cas9 and NGS/GWAS enable the modeling of human congenital diseases at the sub-cellular and molecular resolution.

Zebrafish as a model for human congenital diseases has immense, untapped potential. A thorough comparative analysis of the fish genome and physiology with respect to human is imperative to gain clinical relevance of this model for human diseases. The zebrafish model will be a promising platform for precision medicine initiatives for many rare human disorders. In this issue, we invited articles from different fields of biology which implement zebrafish as a key tool to uncover the secrets of embryonic development and the associated human congenital diseases.

The aim of this issue was to bring together the latest quality articles from researchers working in the area of cell and developmental biology, focused on using zebrafish as a model for human congenital/developmental disorders and rare diseases. The scope of this issue was;

- Modelling human congenital hematopoietic and cardiovascular diseases
- Zebrafish as a model for neurodevelopment and neuronal function
- Zebrafish as a model for metabolic diseases
- Modelling cancer using zebrafish
- Forward and reverse genetic approaches to generate zebrafish disease models
- Technical advances in genetic engineering using zebrafish

In this issue, we showcase how zebrafish has been used as a model to understand the molecular and cellular basis of human congenital disorders such as ciliopathies, cardiovascular syndromes and Fetal Growth Restriction (FGR). To understand the molecular basis of ciliopathies, Lee et al. report a cilia specific injury zebrafish model developing using GAL4/UAS system. The ablation of cilia in this model resulted in cystic kidney, pericardial and periorbital edema. Further, CRISPR-Cas9 based Congenital Nephrotic Syndrome (CNS) disease model was developed by Lee et al. The mutants showed hypoalbuminemia, proteinuria, complete lack of nephrin and glomerular and podocyte defects in a manner akin to human CNS kidney.

A majority of congenital disorders include craniofacial and cardiovascular and heart defects. Sun et al. discuss the Chromodomain-helicase-DNA-binding protein 7 (CHD7) based zebrafish model of CHARGE (Coloboma of the eye, Heart defects, Atresia of the nasal choanae, Retardation of growth and/or development, Genital and/or urinary abnormalities, and Ear abnormalities and deafness) syndrome which exhibit heart abnormalities such as aberrant branching of arteries and associated craniofacial defects. Risato et al. focused to identify signaling mechanisms which regulate FGR related cardiovascular disorders. Transcription profiles of hypoxia induced FGR zebrafish model and human FGR umbilical cord samples reveal dysregulation of Jak/Stat3 and Wnt/β-catenin signaling pathways. Chemical genetic analysis was done to test candidate drugs which target Wnt/β-catenin and Jak/Stat3 pathways to rescue the FGR associated phenotypes in zebrafish model. The authors identify Wnt/β-catenin signaling as a promising FGR marker, poised for detailed pharmacological studies for FGR intervention.

Zebrafish is also commonly used for large, high throughput screening of therapeutic compounds and drugs. Vedder et al. developed a high throughput drug screen of 1,280 compounds to assess developmental toxicity and quantify phenotypic alterations in congenital cardiovascular disorders. The authors developed a Python-based tool to determine cardiac chamber specific parameters like heart rate, arrhythmia and contractility. The candidate compounds showed various phenotypes such as teratogenesis, cardiac contractility defects and atrioventricular block. The platform serves as a novel, open access tool to study cardiac malformations, functions and cardiotoxicity.

To summarize, the zebrafish disease models provide deep mechanistic insights into pathophysiology of the congenital disorders and can be used as screening platforms for improved therapeutic strategies and precision medicine initiatives.

